# Literacy acquisition facilitates inversion effects for faces with full-, low-, and high-spatial frequency: evidence from illiterate and literate adults

**DOI:** 10.3389/fpsyg.2023.1061232

**Published:** 2023-04-24

**Authors:** Qi Yang, Lina Zhang, Changming Chen, Xiaohua Cao

**Affiliations:** ^1^School of Humanities, Tongji University, Shanghai, China; ^2^School of Psychology, Zhejiang Normal University, Jinhua, China; ^3^School of Educational Sciences, Chongqing Normal University, Chongqing, China; ^4^Key Laboratory of Intelligent Education Technology and Application of Zhejiang Province, Zhejiang Normal University, Jinhua, China; ^5^Zhejiang Philosophy and Social Science Laboratory for the Mental Health and Crisis Intervention of Children and Adolescents, Zhejiang Normal University, Jinhua, China

**Keywords:** literacy acquisition, configural face processing, spatial frequency, face inversion paradigm, script system

## Abstract

Previous studies have found that literacy acquisition modulates configural face processing (i.e., holistic and second-order configural processing). However, it remains unclear how literacy acquisition impacts the configural processing indexed by the inversion effect of normal or filtered faces. We asked Chinese illiterate and literate adults to judge whether two sequentially-presented stimuli, including faces, houses (experiment 1), and high- or low-pass filtered faces (experiment 2) were identical. Literate adults outperformed illiterate controls in the upright face and house conditions (experiment 1) and the upright high- and low-pass filtered conditions (experiment 2) but not in the inverted conditions. Notably, the size of an inversion effect (i.e., subtracting inverted accuracy from upright accuracy) was greater among literate adults than that among illiterate adults in both experiments. These findings support that literacy acquisition promotes configural face processing.

## 1. Introduction

Learning to read is a critical milestone in human development. The plasticity of the human brain lays a foundation for which learning to read reshapes the structure and function of the brain organization in processing linguistic materials (such as words) and non-linguistic materials (such as faces) (Dehaene et al., 2015). This study centers on the impact of literacy acquisition on configural face processing, which refers to any phenomenon involving perceiving relations among facial features (Farah et al., 1998).

A recent neural recycling hypothesis claims that learning to read is a great invention over evolutionary history. However, this history is too short for neural areas to have evolved specifically for word processing. To this end, literacy acquisition needs to “invade” other neural areas, typically ones that subserve face and object processing (Dehaene and Cohen, 2007; Dehaene et al., 2010). In line with the theoretical framework, a growing body of empirical studies has supported the association between literacy and face processing (Dehaene et al., 2015; Feng et al., 2022). For example, the left visual word form area (VWFA), an occipitotemporal temporal region preferentially responsive to visual words, is less responsive to faces for literate adults as opposed to illiterate adults (Dehaene et al., 2010). Neural activation of faces in VWFA decreases gradually as the knowledge of letters increases among young children (Cantlon et al., 2010). Early exposure to reading also delays face lateralization indexed by electrophysiological signals in the occipitotemporal areas roughly corresponding to the VWFA in Chinese (Li et al., 2013) and American children (Dundas et al., 2013, 2014). In addition, dyslexic readers show poor performance in both word and face processing (Jozranjbar et al., 2021) and reduce hemispheric lateralization to words and faces compared to normal readers (Gabay et al., 2017). Face recognition impairments are found to be more severe in the bilateral than the unilateral temporo-occipital cortex (Gainotti and Marra, 2011). A left occipital arteriovenous malformation results in pure alexia and prosopagnosia (Liu et al., 2011). These findings suggest that both distinct and overlapping neurocognitive computations subserve the processing of faces and words; and there are both cooperative and competitive interactions regarding the development of visual representations for these two categories (Plaut and Behrmann, 2011).

Despite that prior studies have demonstrated a link between literacy acquisition and face processing, few studies have investigated how literate and illiterate populations may undergo different configural face processing, which is a specific aspect of face processing (Ventura et al., 2013; Cao et al., 2019; Rossion and Lochy, 2021; Yang et al., 2021). The configural face processing is usually measured by different experimental paradigms, such as the face inversion paradigm, the composite face paradigm, and the spatial configuration paradigm (Maurer et al., 2002). Furthermore, despite the limited literature that did explore the relationship between literacy acquisition and configural face processing with the composite face paradigm and spatial configuration paradigm, different experimental paradigms tell different stories regarding cognitive mechanisms underlying configural face processing (Maurer et al., 2002; Richler et al., 2012). Therefore, varying paradigms may be necessarily adopted to understand how literacy affects configural face processing. We explain the differences among the aforementioned paradigms for configural face processing in detail below. The face inversion paradigm taps the first-order configural processing. The features of upright faces are arranged with two eyes above a nose, which is above a mouth (the first-order relationship). The inverted presentation disrupts the first-order configural information (Yin, 1969). The composite paradigm taps the holistic processing, gluing the features into a gestalt, and reflects the failure of selective attention to facial parts (Gauthier and Bukach, 2007). The spatial configuration paradigm instructs the observers to perceive the spatial distances among facial features, which measures the sensitivity to the second-order configural relations (Maurer et al., 2002; Cao et al., 2019).

Recent empirical findings seemed to support the hypothesized link between literacy and configural face processing. For example, with the composite face paradigm, Ventura et al. (2013) found that illiterate adults processed faces and houses more holistically than their literate counterparts. Recently, combining the spatial configuration and the inverted face paradigms, Cao et al. (2019) found that the illiterate were less sensitive to changes in the spatial arrangement among facial features in upright conditions than the literate, but showed comparable spatial sensitivity in inverted conditions to the literate. These findings suggested that literacy acquisition could reshape configural face processing.

However, unlike the first two experimental paradigms, the face inversion paradigm has not been widely used to investigate the literacy effect on configural face processing. Moreover, Cao et al. (2019) concentrated on the spatial configuration of faces and presented two faces or houses simultaneously. Many studies have demonstrated that a stimulus’s presentation type (i.e., simultaneous vs. sequential) could generate distinct cognitive processes or processing strategies (Hole, 1994; Richler et al., 2009; Li and Cao, 2017). For instance, the simultaneous presentation may pose less memory load for observers than the sequential presentation and encourage participants to utilize part-based, feature-matching strategies or image-matching strategies (Hole, 1994; Richler et al., 2009), while sequential presentation may promote participants to deal with faces holistically, as well as reduce the participants’ tendency to compare the local features of faces (Yang and Schwaninger, 2010). Thus, the first goal of the present research is to investigate whether the literacy effect on face inversion effect could still be observed when paired faces are sequentially presented.

Furthermore, previous literature has investigated the literacy effect on configural face processing with full-spatial-frequency faces. Spatial frequency refers to several cycles per degree of visual angle or several cycles per image (Morrison and Schyns, 2001), which is an essential determinant of visual processing. High spatial frequency is generally linked with featural and local information, whereas low spatial frequency is with coarse and global information (Sergent, 1986; Boutet et al., 2003; Cheung et al., 2008). Previous studies debated whether only low-spatial frequencies or both low- and high-spatial frequencies subserve configural face processing (Goffaux and Rossion, 2006; Cheung et al., 2008). For instance, Goffaux and Rossion (2006) found that the holistic face processing was driven by the low spatial frequency not by the high spatial frequency in the partial composite face paradigm, while Cheung et al. (2008) showed that holistic face processing was supported by both low spatial frequency and high spatial frequency in the complete composite face paradigm. In the composite face paradigm, participants are required to attend the top (or bottom) half of a composite face (relevant part) while ignoring the bottom (or top) half (irrelevant part) and judge whether the relevant half of the study face is identical to that of the test face. In the partial composite paradigm, the irrelevant parts of both study and test faces are always different, while in the complete paradigm, the irrelevant parts of both study and test faces are either same or different. Moreover, a previous study demonstrated that, relative to poor readers, good readers showed enhanced sensitivity to low-spatial frequencies ranging from 2 to 6 cyc/deg, and no differences were observed in sensitivity to high-spatial-frequencies among poor and good readers ranging from 64 to 128 cyc/deg (Patching and Jordan, 2005). Converging these findings, we thus want to ask whether literacy acquisition could drive individuals to adopt certain spatial frequencies to support configural face processing. To the best of our knowledge, it remains unexplored how spatial frequency modulates the literacy effect on configural face processing. Therefore, the second purpose of our research is to investigate whether the literacy effect on face inversion processing could also be observed in those faces with a specific range of spatial frequencies preserved.

To sum up, we ran two experiments to further the understanding of the literacy effect on configural face processing. We compared face and house processing in the first experiment between literate and illiterate adults. According to the neural recycling hypothesis, one main prediction is that the literate and illiterate should demonstrate differential patterns in the face inversion effect. In particular, based on a previous study (Cao et al., 2019), we predict that literate adults would show a more significant recognition performance than illiterate adults in upright rather than inverted faces and even houses when these stimuli are presented sequentially. In the second experiment, we want to further explore whether literacy acquisition improved face inversion processing in distinct spatial frequencies. If in line with the findings from Cheung et al. (2008), the present study should hypothesize that literacy acquisition enhances the configural processing of faces in both spatial frequencies. If in line with the findings from Goffaux and Rossion’s and Patching and Jordan’s study (Patching and Jordan, 2005; Goffaux and Rossion, 2006), the present study should expect that the literacy effect could only be found in the low-spatial-frequency condition. More importantly, a larger face inversion effect size, indexed by subtracting inverted accuracy from upright accuracy or by subtracting upright response time from inverted response time, is expected for literates than for illiterates in both experiments.

## 2. Experiment 1

### 2.1. Participants

We recruited 15 illiterate (14 female) and fifteen literate adults (14 female) from the same rural village in central China. Illiterate adults had an average age of 64.40 years old (range: 53−77 years; *SD* = 6.50 years), and the average age of literate adults was 61.00 years old (range: 56−68 years; *SD* = 3.35 years). The G-Power 3.1 (Faul et al., 2007) was used to assess the required sample sizes based on a prior study (Cao et al., 2019), in which the effect size (η*_*p*_*^2^ = 0.21) of interaction between Literacy and Orientation was applied to the present experiment. A power analysis showed that a sample size of 5 in one group would be required at the 0.05 alpha level with 90% power. Thus, we had enough sample size to detect this effect size. The illiterate adults were identified by the Grade one literacy questionnaire (Wang and Tao, 1993), the Mini-Mental State Examination (MMSE, Wu et al., 2002), and the Word-color Stroop task. These illiterates were those who could not recognize Chinese characters in the one, two, and three lists or less than five Chinese characters in succession in the Grade One literacy questionnaire (Wang and Tao, 1993; Cao et al., 2019), and could pass the test of MMSE (the score is above 14). We used the scores of MMSE to ensure that none of the participants experienced cognitive impairment, and used the scores of the Word-color Stroop task to identify that the illiterate participants indeed could not recognize common Chinese characters. These critical cognitive abilities measured in these tasks are considered core consequences of literacy acquisition, which has been used to distinguish between illiterate and literate individuals (Cao et al., 2019). All of them were orally reported as right-handed, with normal or corrected-to-normal vision. None of the participants has a history of mental illness or cognitive impairment. All participants and their relatives received verbal or written informed consent, and the ethical committee at Zhejiang Normal University approved the study.

### 2.2. Stimuli

**FIGURE 1:**
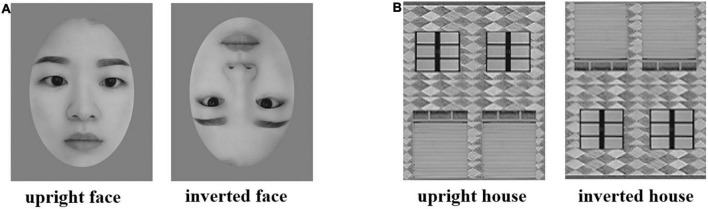
Panels **(A,B)** are examples of the upright and inverted face and house stimuli in Exp. 1.

The original materials were twenty Chinese adults’ upright face pictures with neutral expressions (half of them were male faces) (Cao et al., 2016, 2019; Zhang et al., 2021). Contour features, such as hair and ears, were cropped out using Photoshop (PS), and the isolated face pictures were put against a neutral gray background. Twenty upright houses were used as control materials, taken from the local village where participants lived (Cao et al., 2019). The production process of the upside house pictures was the same as that of the face pictures. The lightness and brightness of the pictures were generally identical. We used the software Adobe Photoshop CS5 (San Jose, CA) to adjust the lightness and brightness of visual stimuli and kept two types of visual stimuli similar in lightness and brightness (houses: 124 ± 19.58 cd/m^2^; full-spatial frequencies faces: 147 ± 1.84 cd/m^2^). We rotated the upright face/house pictures 180 degrees to make the inverted face/house pictures (see Figure 1). The visual angle of each picture is 4.0^°^ × 6.2^°^. The experiment was carried out on Lenovo’s 14 inches G470 laptop with a screen resolution of 1024 × 768 (Zhang et al., 2018). The experiment was run based on the E-prime 1 software^1^.

### 2.3. Procedure

**FIGURE 2:**
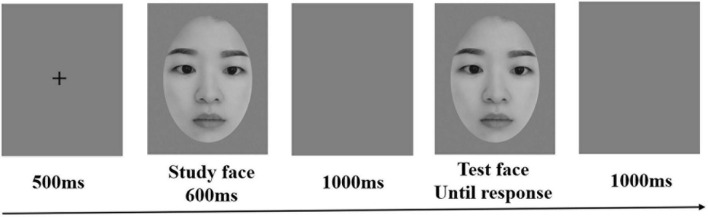
The example of the upright-face conditions for one trial. Participants are required to judge whether the study face and the following test face are identical.

To begin with, a 500 ms black fixation cross appeared against a white background at the center of the screen, followed by a study stimulus for 600 ms. Then, a blank gray screen was displayed for 1,000 ms, followed by a test stimulus, which was displayed in the same orientation as the study stimulus. The test stimulus would disappear as soon as the participant initiated the response (see Figure 2). The task of the participants is to judge whether the study stimulus and the test stimulus were identical as quickly and accurately as possible. Half of the participants pressed “A” for “same” or “L” for “different”; for the other half, the key response was counterbalanced. The inter-trial interval was 1,000 ms. Participants completed 16 practice trials before the formal experiment. Only when the accuracy of the practice procedure was higher than 75% could they be allowed to start the formal experiment. Otherwise, they needed to complete the practice procedure again and again. The formal experiment consisted of four blocks with a total of one hundred and sixty trials, and each block had twenty trials for each condition (i.e., upright and inverted conditions).

### 2.4. Data analysis

We used SPSS 22.0 software^2^ to analyze the accuracy and response time with a 2 (Literacy: illiterate vs. literate) × 2 (Stimulus Category: face vs. house) × 2 (Orientation: upright vs. inverted) mixed analysis of variance (ANOVA). In this model, we took Stimulus Category and Orientation as within-subject factors and Literacy as the between-subjects factor. We also performed independent sample *t*-tests to examine the difference in the size of the face inversion effect between literate and illiterate groups when the interaction of Literacy and Orientation was significant. We deleted 1.2% of trials accounting for all correct trials whose response times were less than 300 ms or longer than 3,000 ms (Cao et al., 2019).

### 2.5. Results

#### 2.5.1. Basic information

The ages were matched, *t*(28) = −1.57, *p* = 0.140, *d* = 0.59, but the years of education differed significantly between both groups, *t*(14.57) = 12.17, *p* < 0.001, *d* = 4.60 (*M*_literate_ = 8.67 ± 0.69 years vs. *M*_illiterate_ = 0.21 ± 0.10 years).

#### 2.5.2. Analysis of accuracy

A repeated measures ANOVA showed the main effects of Stimulus Category with higher accuracy for houses (0.94) than faces (0.77), *F*(1, 28) = 122.34, *p* < 0.001, η*_*p*_*^2^ = 0.81, Orientation with higher accuracy for upright (0.89) than inverted condition (0.82), *F*(1, 28) = 41.11, *p* < 0.001, η*_*p*_*^2^ = 0.60, and Literacy with higher accuracy for literates (0.88) than illiterates (0.83), *F*(1, 28) = 6.21, *p* = 0.019, η*_*p*_*^2^ = 0.18.

**FIGURE 3:**
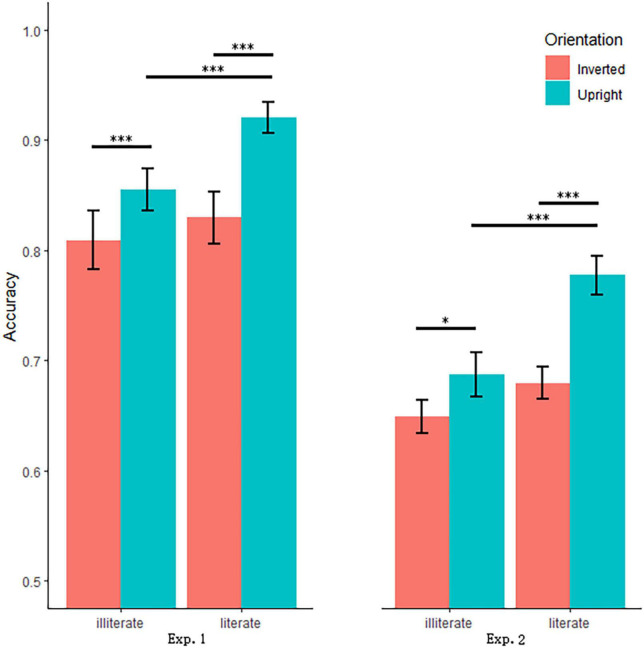
Bar plot with mean accuracy and standard errors as a function of Literacy and Orientation in experiment 1 **(left)** panel and in experiment 2 **(right)** panel. Error bars indicated standard errors. * Indicates *p* < 0.05, and ^***^ indicates *p* < 001.

The analysis also found that Literacy interacted with Orientation, *F*(1, 28) = 4.35, *p* = 0.046, η*_*p*_*^2^ = 0.13. The simple effect analysis revealed that accuracy was higher for literate (0.92) than illiterate adults (0.86) in upright condition, *t*(28) = 3.61, *p* = 0.001, *d* = 1.36, and that accuracy was comparable between illiterates (0.81) and literates (0.83) in inverted condition, *t*(28) = −0.92, *p* = 0.365, *d* = 0.35. Another direction of this interaction showed that accuracy was higher in upright than inverted conditions for literates, *t*(14) = 4.61, *p* < 0.001, *d* = 2.46, and illiterates, *t*(14) = 5.58, *p* < 0.001, *d* = 2.98, separately (see Figure 3). Independent-sample *t*-test demonstrated a larger size of inversion effect in literate adults than illiterate controls, *t*(28) = 2.09, *p* = 0.046, *d* = 0.79.

The interaction of Stimulus Category by Literacy was also significant, *F*(1, 28) = 8.50, *p* = 0.007, η*_*p*_*^2^ = 0.23. Separate analyses for the two stimuli revealed that as for faces, accuracy was lower for illiterates (0.73) than for literates (0.81), *t*(28) = −3.15, *p* = 0.004, *d* = 1.19, but as for houses, there was similar performance between illiterates (0.94) and literates (0.94), *t*(28) = 0.05, *p* = 0.961, *d* = 0.02.

The analysis also yielded an interaction between Stimuli Category and Orientation, *F*(1, 28) = 38.17, *p* < 0.001, η*_*p*_*^2^ = 0.58, with higher accuracy of upright (0.83) than inverted faces (0.71), *t*(29) = 7.14, *p* < 0.001, *d* = 2.65), and with similar performances between upright (0.94 ± 0.04) and inverted houses (0.93), *t*(29) = 1.19, *p* = 0.244, *d* = 0.44. We found an insignificant three-way interaction, *F*(1, 28) = 0.28, *p* = 0.601, η*_*p*_*^2^ = 0.01.

#### 2.5.3. Analysis of response time

A repeated measures ANOVA showed the main effects of Stimulus Category with an overall faster response of houses (778 ms) than faces (956 ms), *F*(1, 28) = 42.03, *p* < 0.001, η*_*p*_*^2^ = 0.60, and Orientation with the quicker response speed of upright (849 ms) than inverted stimuli (885 ms), *F*(1, 28) = 14.16, *p* = 0.001, η*_*p*_^2^* = 0.34.

The main effect of Literacy was not significant, *F*(1, 28) = 2.64, *p* = 0.115, η*_*p*_*^2^ = 0.09. In addition, neither two-way interactions (Stimulus Category by Orientation, *F*(1, 28) = 0.95, *p* = 0.338, η*_*p*_^2^* = 0.03; Stimulus Category by Literacy, *F*(1, 28) = 3.41, *p* = 0.075, η*_*p*_^2^* = 0.11; Orientation by Literacy, *F*(1, 28) = 0.02, *p* = 0.891, η*_*p*_^2^* < 0.01) nor three-way interaction, *F*(1, 28) = 0.30, *p* = 0.590, η*_*p*_^2^* = 0.01, was found.

The present experiment revealed that recognition accuracy was higher for literates than for illiterates in upright rather than inverted faces, consistent with the previous study (Cao et al., 2019, experiment 1). The effect also extends to house processing, similar to previous findings (Cao et al., 2019, experiment 2). Moreover, the present study also revealed that literate adults showed a larger inversion effect size than illiterate adults. In sum, the experiment provided insights into the close link between literacy and non-word processing. To gain a reliable literacy effect on configural face processing, we tried to extend the effect to these faces filtered with distinct spatial frequencies in experiment 2.

## 3. Experiment 2

### 3.1. Participants

We recruited eleven illiterate (ten female) and eleven literate adults (11 female) from a rural area of central China. Note that eight illiterate adults (one male) and all literate adults participated in Experiment 1 because illiterate adults were not readily available in the region where the study was conducted. Illiterate adults had an average age of 63.27 years old (range: 53−69 years; *SD* = 4.96 years), and the average age of literate adults was 61.00 years old (range: 56−68 years; *SD* = 3.90 years). The G-Power 3.1 was used to assess the required sample sizes in experiment 2. In this analysis, the mean effect size (η*_*p*_*^2^ = 0.17) of Cao et al. and experiment 1 in the present study was adopted to calculate the sample size, which demonstrated that a sample size of six in one group would be required at 0.05 alpha level with 90% power. Thus, the sample size used in the present experiment is necessary to detect this effect size. All of them were right-handed native Chinese with normal or corrected-to-normal vision. All participants and relatives received verbal or written informed consent, and the ethical committee at Zhejiang Normal University approved the study. The screening of subjects was the same as in experiment 1.

### 3.2. Stimuli

**FIGURE 4:**
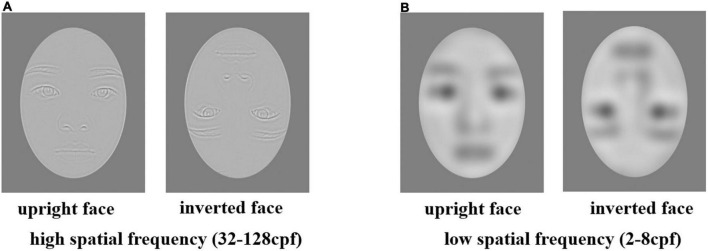
Panel **(A)** is an example of a pair of upright and inverted faces in high spatial frequency conditions. Panel **(B)** shows the example of a pair of upright and inverted faces in low spatial frequency conditions.

The face stimuli of experiment 2 are all from experiment 1. MATLAB software^3^ was used to filter all the face materials with low-spatial frequency (2−8 cycles per face) and high-spatial frequency (32−128 cycles per face) (Goffaux and Rossion, 2006; Cheung et al., 2008). Figure 4 shows an exemplar stimulus for each condition.

### 3.3. Procedure

The experiment procedure is the same as experiment 1.

### 3.4. Data analysis

We used SPSS 22.0 software (see text footnote 2) to analyze the accuracy and response time with a 2 (Literacy: literate vs. illiterate) × 2 (Spatial Frequency: high vs. low) × 2 (Orientation: upright vs. inverted) ANOVA. In this model, we took Spatial Frequency and Orientation as within-subjects factors and Literacy as the between-subjects factor. In addition, we performed independent sample *t*-tests to examine differences in the size of the face inversion effect between literate and illiterate groups when the interaction of Literacy and Orientation was significant. We deleted 5.1% of trials accounting for all correct trials whose response times were less than 300 ms or longer than 3,000 ms.

### 3.5. Results

#### 3.5.1. Basic information

The average ages were matched, *t*(20) = −1.50, *p* = 0.166, *d* = 0.90, but the average years of education differed between both groups, *t*(10) = 10.06, *p* < 0.001, *d* = 4.60 (*M*_literate_ = 8.45 ± 2.69 years vs. *M*_illiterate_ = 0.18 ± 0.41 years).

#### 3.5.2. Analysis of accuracy

A repeated measures ANOVA revealed the main effects of Spatial Frequency with higher accuracy for low-spatial-frequency condition (0.74) than high-spatial-frequency condition (0.66), *F*(1, 20) = 21.50, *p* < 0.001, η*_*p*_^2^* = 0.52, Orientation with higher accuracy for upright (0.73) than inverted conditions (0.66), *F*(1, 20) = 34.96, *p* < 0.001, η*_*p*_^2^* = 0.64, and Literacy with higher accuracy for literates (0.73) than illiterates (0.67), *F*(1, 20) = 10.19, *p* = 0.005, η*_*p*_^2^* = 0.34.

There was only an interaction between Literacy and Orientation, *F*(1, 20) = 6.68, *p* = 0.018, η*_*p*_^2^* = 0.25. Separate analyses for the two orientations revealed that there was a significant distinction between illiterates (0.69) and literates (0.78) for upright faces, *t*(20) = -4.00, *p* = 0.001, *d* = 1.79, but not between illiterates (0.65) and literates (0.68) for inverted faces, *t*(20) = −1.40, *p* = 0.177, *d* = 0.63. Another direction of this interaction showed that the accuracy was higher for the upright condition than for the inverted condition for both groups (literates: *t*(10) = 6.04, *p* < 0.001, *d* = 3.82; illiterates: *t*(10) = 2.34, *p* = 0.041, *d* = 1.48) (see Figure 3). The independent sample *t*-test showed that the inversion effect size was larger in literate adults than in illiterate controls, *t*(20) = 2.59, *p* = 0.018, *d* = 1.16.

We found neither the interaction between Spatial Frequency and Orientation, *F*(1, 20) = 2.54, *p* = 0.127, η*_*p*_^2^* = 0.11, nor the interplay between Literacy and Spatial Frequency, *F*(1, 20) = 0.02, *p* = 0.877, η*_*p*_^2^* = 0.001. Also, there was no significant triple interaction among Literacy, Orientation, and Spatial Frequency, *F*(1, 20) = 0.12, *p* = 0.737, η*_*p*_^2^* = 0.01.

#### 3.5.3. Analysis of response time

The analysis revealed the main effect of Spatial Frequency with a quicker response speed of low-spatial-frequency faces (964 ms) than high-spatial-frequency faces (1,161 ms), *F*(1, 20) = 12.27, *p* = 0.002, η*_*p*_^2^* = 0.38. Other main effects were not significant.

There was only one interaction between Literacy and Spatial Frequency, *F*(1, 20) = 6.46, *p* = 0.019, η*_*p*_^2^* = 0.24. Separate analyses for the two kinds of Spatial Frequency revealed that the difference between illiterates (1,027 ms) and literates (1,295 ms) was marginally significant for high-spatial-frequency faces, *t*(20) = −2.02, *p* = 0.057, *d* = 0.90, but significant distinction between illiterates (973 ms) and literates (956 ms) for low-spatial-frequency faces was not reached, *t*(20) = 0.177, *p* = 0.861, *d* = 0.08.

We revealed neither the interaction between Spatial Frequency and Orientation, *F*(1, 20) = 0.46, *p* = 0.507, η*_*p*_^2^* = 0.02, nor the interplay of Literacy and Orientation, *F*(1, 20) = 0.97, *p* = 0.336, η*_*p*_^2^* = 0.05. The three-way interaction of Literacy by Orientation by Spatial Frequency was not significant, *F*(1, 20) < 0.01, *p* = 0.957, η*_*p*_^2^* < 0.01.

## 4. Discussion

The present study aimed to examine the literacy effect on the first-order configural face processing and conducted two experiments to compare the face recognition performance and the size of the inversion effect between literate and illiterate adults when these stimuli were presented consecutively. In experiment 1, we found that illiterate adults showed lower accuracy than literate controls for upright faces but comparable accuracy for inverted faces. This literacy effect also extended to house processing. In experiment 2, we also observed a two-way interaction between Literacy and Orientation, similar to experiment 1. However, high-and low-spatial frequencies did not modulate the interaction between Literacy and Orientation in face processing. In addition, the illiterate responded faster to faces with high spatial frequency, rather than faces with low spatial frequency, as compared with the literate. Notably, both experiments showed that the inversion effect size was more prominent in literate than illiterate adults. These findings further highlighted the link between literacy acquisition and configural face processing.

### 4.1. Literacy acquisition promotes the first-order configural processing

Consistent with the neural recycling hypothesis (Dehaene and Cohen, 2007; Dehaene et al., 2010), which claims that literacy acquisition “invades” these cortexes typically for processing faces or other objects, the present study showed a larger inversion effect for faces and houses in literate adults compared with illiterate control. Moreover, the literate performed better in recognizing upright faces and houses presented sequentially than the illiterate. The present study, combined with a prior study in which paired faces were presented simultaneously (Cao et al., 2019), suggested that the way of stimuli presentation (simultaneous vs. sequential) seems not to change the outcome of literacy facilitation effect on face inversion effect. However, these two presentations may tax different cognitive loads (Menon et al., 2015) and require distinct processing strategies (Hole, 1994; Richler et al., 2009). The previous literature demonstrated that simultaneous presentation required less cognitive effort compared with sequential presentation; simultaneous presentation facilitated participants to process faces holistically, and even decreased the preference for the use of the local features (Yang and Schwaninger, 2010), while participants in the sequential presentation may employ memory-based-implicating strategies or the outcome-of-an-attention strategies. In sum, the findings suggested that the literacy effect on the inversion effect for faces was stable.

In addition, the literacy effect on face processing also extended to non-facial stimuli (i.e., house) processing, which suggested that the literacy effect might be domain-general, supporting the neural recycling hypothesis. More precisely, these original cortexes that learning to read “invades” may subserve domain-general cognitive ability.

### 4.2. Literacy acquisition may attenuate response speed to high spatial frequency

In the present study, we also found partial evidence that the illiterate participants reacted faster to faces with high spatial frequency than the literate participants, while a comparable response speed on faces with low spatial frequency was observed between illiterate and literate adults. The findings seem to suggest that literacy acquisition attenuated the processing speed to the facial information conveyed by the high spatial frequency. In other words, the illiterate seem to be inclined to employ the featural information to process faces as compared with the literate, given that the high spatial frequency transmits more features or local information. These findings were not inconsistent with the view that literacy acquisition improved configural face processing (such as Cao et al., 2019). Additionally, Patching and Jordan (2005) found that good readers showed higher sensitivity to low spatial frequency as compared with poor readers. Although poor readers and illiterate adults had similarities in terms of reading, they were essentially different groups. To sum up, combined with these studies mentioned above, the present study provided insights into the complex relationship between literacy and the sensitivity to distinct spatial frequencies.

Finally, we found that the literacy effect on face processing was not modulated by spatial frequency. This is consistent with previous research, which shows that low and high spatial frequency evoked holistic face processing in a complete composite face paradigm (Cheung et al., 2008). These studies suggested that literacy acquisition seemed not to drive different sensitivities to spatial frequency to support configural face processing.

### 4.3. Two possibilities concerning the literacy effect on configural face processing

The two experiments in the present study both showed the facilitated face inversion effect of literacy, (partially) consistent with the findings of Cao et al. (2019) and Ventura et al. (2013). Cao et al. (2019) used the spatial configuration task and showed that illiterate adults were less sensitive to the relationships between facial features than literate controls in upright rather than inverted faces. However, Ventura et al. (2013) found that literacy acquisition decreased the holistic processing indexed by the composite face effects. Two possibilities could account for the inconsistent result patterns between Ventura et al. (2013) and the present study. One obvious possibility may be associated with the differences in these experimental paradigms (composite face paradigm and face inversion paradigm). The composite face paradigm reflects the failure of selective attention to facial parts, whereas the face inversion paradigm demonstrates the disruption of the typical arrangement of facial features. Different experimental paradigms could tap into the different dimensions of configural face processing (Maurer et al., 2002; Richler et al., 2012), given the complex relationships among different paradigms (Teunisse and De Gelder, 2003; Wang et al., 2012; DeGutis et al., 2013; Rezlescu et al., 2017). Therefore. future studies are encouraged to perform a face inversion task among western literate and illiterate adults or to conduct a composite face task in Chinese illiterates and literates.

An alternative possibility is an attention-shaped-by-language hypothesis (Yang et al., 2021), which suggests that language shapes how its user deploys the attentional resources to the visual processing of words, given the extensive and longstanding experience with the visual features of words. In the current case, literates acquired Portuguese scripts in Ventura et al.’s (2013) study, whereas literate adults acquired Chinese scripts in the present study and Cao et al.’s (2019)study. Chinese characters may be special cases as they possess a highly non-linear visual complexity of shape (Huang et al., 2004), while Portuguese words are composed of basic letters and are linear. Long-term and extensive exposure to the differences in visual features between Chinese and Portuguese scripts may induce distinct processing demands for Chinese compared to Portuguese scripts and further transfer to the processing of configural faces. Recently, Ma et al. (2022) showed that N170 amplitudes elicited by naturalistic and Mooney faces were larger for Chinese children than for German children, which suggested superior holistic processing in Chinese children. Using the Framed-Line Test in Portugal and Thailand, Ventura et al. (2008) found that Portuguese literates showed higher accuracy in the absolute task (reflecting the analytic processing) compared to the relative task (reflecting the holistic processing). However, Portuguese illiterates and ex-illiterates showed higher accuracy in the relative task than in the absolute task. However, all Thai groups showed consistently higher accuracy on the relative task than on the absolute task. The attention-shaped-by-language hypothesis seems to account for the domain-general mechanism underlying the literacy effect on object inversion processing (such as faces and houses). In a word, these findings demonstrated that schooling/literacy imposed different impacts on the participants with distinct script systems. Future studies may find it worthwhile to explore new experimental designs to examine the attention-shaped-by-language hypothesis.

### 4.4. Implications and limitations

The current data adds ingredients to the theoretical development of the relations between literacy and face processing by examining the first-order configural processing (i.e., face inversion effect) between Chinese literate and illiterate adults. Following the neural recycling hypothesis, previous studies mainly centered on the correlations between face processing and word processing, or between face processing and the amount of literacy. Recently, few studies (including the present study) with different paradigms of configural face processing shed light on how literacy acquisition affects configural face processing, particularly in different script systems (Ventura et al., 2013; Cao et al., 2019). Combined with the existing findings, the present study also proposed that the attention-shaped-by-language hypothesis could account for the cognitive mechanisms underlying literacy acquisition on configural face processing. Moreover, the present study also has some implications for further studies, which should be conducted by recruiting those literate and illiterate adults trained with different script systems.

Some limitations may be considered in the present study. First, socialization may reduce the credibility of making causal inferences. Participants in both groups were matched as closely as possible on age and gender, but they may differ in the amount of their exposure to faces. For instance, the literate may experience more distinct faces than the illiterate when attending regular school (Cao et al., 2019). They have more access to a broader range of people after acquiring literacy. The experience of faces may lead to differential effects on configural face processing (Balas and Saville, 2015).

Second, previous studies demonstrated that females performed better face perception and cognition than males (Sommer et al., 2013). However, in the present study, almost illiterate adults were female (only one male) because males had more opportunities to attend school in China in the 20th century. Therefore, we shall be cautious to generalize the current findings to male illiterate adults, or other populations.

## 5. Conclusion

The present study concluded that the literate promoted face recognition when upright faces were presented. The effect also extends to different stimuli (i.e., houses) and faces with distinct spatial frequencies. These findings shed insight on the close association between literacy acquisition and configural face processing.

## Data availability statement

The raw data supporting the conclusions of this article will be made available by the authors, without undue reservation.

## Ethics statement

The studies involving human participants were reviewed and approved by the Ethics Committee of Zhejiang Normal University. The patients/participants provided their written informed consent to participate in this study. Written informed consent was obtained from the individual(s) for the publication of any identifiable images or data included in this article.

## Author contributions

QY, CC, and XC: conceptualization. QY and LZ: data curation, formal analysis, and visualization. XC: funding acquisition and project administration. QY: investigation. QY and XC: methodology. QY, LZ, and CC: roles/writing—original draft. All authors: writing—review and editing.

## Footnotes

1
https://pstnet.com/

2
https://www.ibm.com/

3
https://www.mathworks.com/
