# Supplementary material for: Literacy acquisition facilitates inversion effects for faces with full-, low-, and high-spatial frequency: evidence from illiterate and literate adults
Source: Front Psychol. 2023 Apr 24;14:1061232. doi: 10.3389/fpsyg.2023.1061232 (PMC10164973; doi:10.3389/fpsyg.2023.1061232)
Supplement: Supplementary file 1 [file Data_Sheet_1.docx]

**Supplemental information**

**1 Tools**

First, the Standardized Chinese Characters Recognition Test was used to assess illiterates’ literacy (Wang and Tao,1993, see Fig. S1). These Chinese characters in the first three groups are used only to screen participants, given the limited amount of literacy. Moreover, we enlarged each Chinese character and presented it on one page. These people were labeled illiterate if they could not identify more than five Chinese characters in succession in the first three groups.

Secondly, the Mini-Mental State Examination was used to screen the cognitive disorders of individuals, especially the elderly (Wu et al., 2002). The total score on the scale is 30. These participants may be likely to suffer from Alzheimer’s disease if the score of illiterate participants is lower than 14 and the score of literate participants is lower than 19, who are not allowed to participate further in the experiment.

Thirdly, to prevent some individuals from deliberately concealing their vocabulary and educational background, the word-color Stroop paradigm was used to examine whether they are illiterate. In the test, if participants could pronounce the word in other colors more than two times, they would be excluded from the present study.

**References**

Wu, C., Zhou, D., & Peter, C., Fan, J. H., & Qiao, Y.L. (2002). Neuropsychological and Functional Study in Screening Alzheimer Disease in Henan Province. *Chinese Mental Health Journal,16*(9), 587-589.

Fig. S1 The literacy questionnaire used to screen the illiterate.


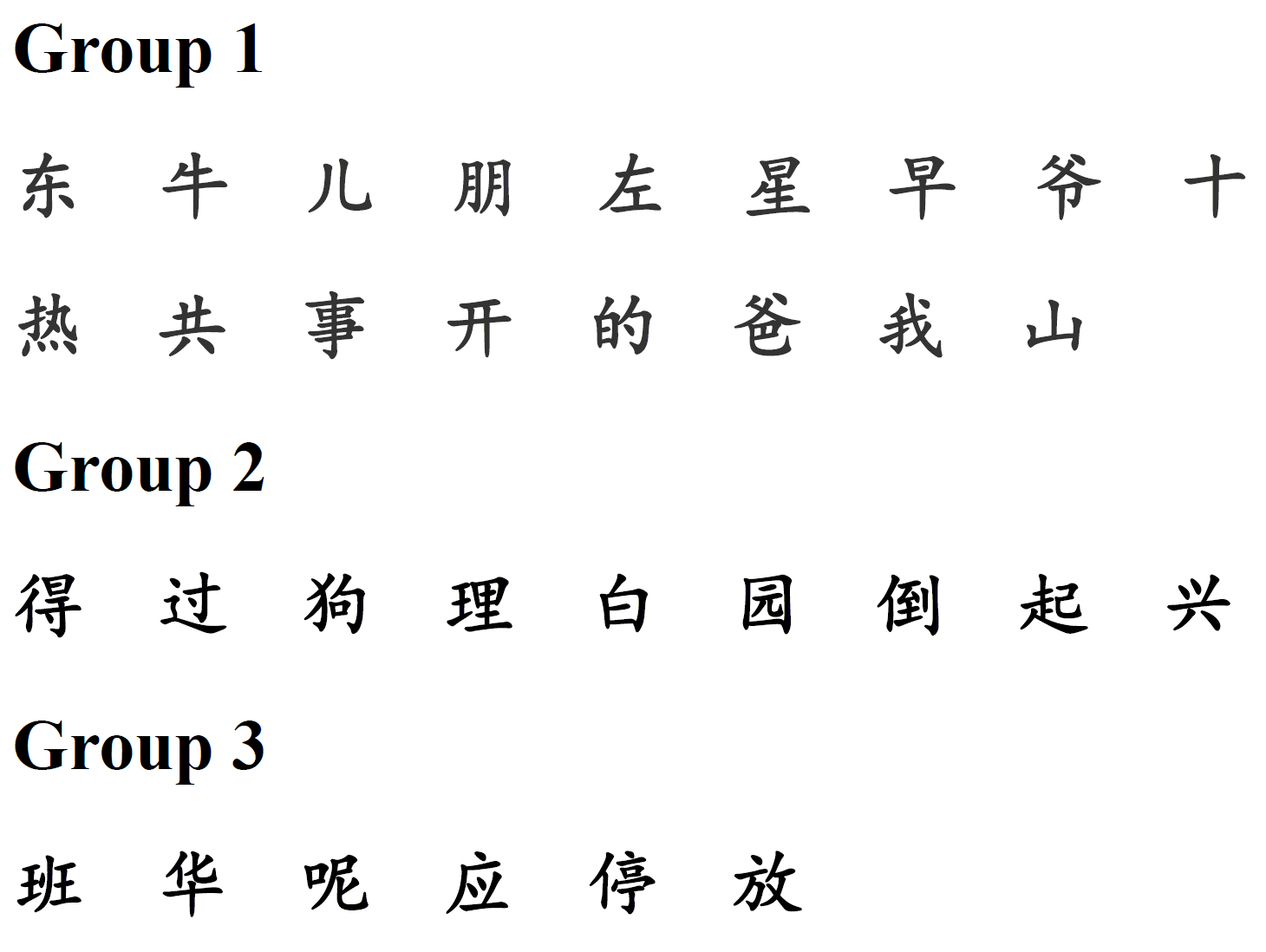


Fig. S2


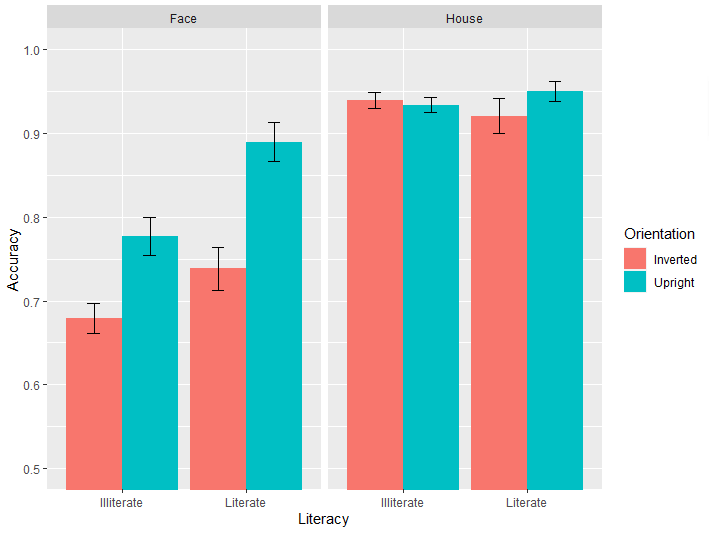


Fig. S2 Bar plot with mean accuracy and standard errors as a function of Literacy stimuli category, and Orientation.
